# Initial Antituberculous Regimen with Better Drug Penetration into Cerebrospinal Fluid Reduces Mortality in HIV Infected Patients with Tuberculous Meningitis: Data from an HIV Observational Cohort Study

**DOI:** 10.1155/2013/242604

**Published:** 2013-08-20

**Authors:** Gerardo Alvarez-Uria, Manoranjan Midde, Raghavakalyan Pakam, Praveen Kumar Naik

**Affiliations:** Department of Infectious Diseases, Bathalapalli Rural Development Trust Hospital, Kadiri Road, Anantapur District, Bathalapalli 515661, Andhra Pradesh, India

## Abstract

Tuberculous meningitis (TM) is the deadliest form of tuberculosis. Nearly two-thirds of HIV infected patients with TM die, and most deaths occur within one month. Current treatment of TM involves the use of drugs with poor penetration into the cerebro-spinal fluid (CSF). In this study, we present the mortality before and after implementing a new antituberculous regimen (ATR) with a higher drug penetration in CSF than the standard ATR during the initial treatment of TM in an HIV cohort study. The new ATR included levofloxacin, ethionamide, pyrazinamide, and a double dose of rifampicin and isoniazid and was given for a median of 7 days (interquartile range 6–9). The new ATR was associated with an absolute 21.5% (95% confidence interval (CI), 7.3–35.7) reduction in mortality at 12 months. In multivariable analysis, independent factors associated with mortality were the use of the standard ATR versus the new ATR (hazard ratio 2.05; 95% CI, 1.2–3.5), not being on antiretroviral therapy, low CD4 lymphocyte counts, and low serum albumin levels. Our findings suggest that an intensified initial ATR, which likely results in higher concentrations of active drugs in CSF, has a beneficial effect on the survival of HIV-related TM.

## 1. Introduction

In 2011, there were 8.7 million incident cases of tuberculosis (13% of them in HIV infected patients) and 1.4 million deaths from tuberculosis (30% of them in HIV infected patients) [1]. With 25% mortality in non-HIV infected patients and 67% in HIV infected patients, tuberculous meningitis has the highest mortality among all forms of tuberculosis [2]. Moreover, tuberculous meningitis is more common in HIV infected patients and can comprise up to 19% of all cases of HIV-related tuberculosis [3, 4].

Currently, treatment of tuberculous meningitis involves the same drugs and doses as other forms of tuberculosis [5–7]. While isoniazid and pyrazinamide have good cerebrospinal fluid (CSF) penetration, rifampicin concentration in CSF may not reach the minimal inhibitory concentration for tuberculosis, and ethambutol and streptomycin have poor CSF penetration [2, 8]. Among second line drugs, levofloxacin, ethionamide and cycloserine have good penetration in CSF [8–10]. In a phase 2 randomized controlled trial investigating the safety of moxifloxacin and a higher intravenous dose of rifampicin during the first two weeks of treatment of tuberculous meningitis, the use of a higher dose of rifampicin was associated with a survival benefit [11]. These data suggest that increasing the CSF penetration of the initial treatment of tuberculous meningitis could potentially reduce its mortality.

The majority of deaths due to tuberculous meningitis occur within one month [4, 12]. Therefore, interventions to reduce mortality should be performed during the early stages of treatment. In this study, we present the mortality before and after implementing a new antituberculous regimen (ATR) with a higher drug penetration in CSF than the standard regimen during the initial treatment of HIV-related tuberculous meningitis.

## 2. Patients and Methods

### 2.1. Setting

This study was performed in the district of Anantapur, India. Rural Development Trust (RDT) is a nongovernmental organization that provides free medical care to HIV infected patients. The Vicente Ferrer HIV Cohort Study (VFHCS) is an open cohort study of all HIV infected patients who have attended RDT hospitals [13, 14]. Since September 2009, clinical information of the patients has been collected prospectively. 

For this study, we selected all cases of tuberculous meningitis diagnosed from 6 June 2010 to 1 April 2013 in HIV infected adults living in Anantapur who were admitted to RDT Hospital Bathalapalli. The selection of patients from the database was executed on 27 May 2013. Patients who did not meet the proposed criteria for definite, probable, or possible tuberculous meningitis were excluded from the analysis [15]. Bacterial culture and cryptococcus antigen tests were performed on CSF to rule out other causes of meningitis.

### 2.2. Treatment

All patients were admitted to the hospital. Before 29 January 2012, patients were treated with the standard oral once daily ATR, that is, isoniazid 300 mg, rifampicin 450 mg, ethambutol 800 mg, and pyrazinamide 1500 mg. After 29 January 2012, patients received a new oral once daily ATR, including isoniazid 600 mg, rifampicin 900 mg, pyrazinamide 1500 mg, levofloxacin 750 mg, and ethionamide 750 mg. Therefore, we doubled the dose of rifampicin and isoniazid, and ethambutol, a drug with poor CSF penetration, was substituted by levofloxacin and ethionamide, which have better CSF penetration [16]. Although isoniazid has good CSF penetration, its dosage was increased due to its bactericidal effect during the first days of treatment [17]. The new ATR was given only during the admission, so once a clinical improvement was observed, patients who received the new ATR were discharged with the standard ATR. After discharge, patients from both groups were treated for at least nine months according to the Indian guidelines for tuberculosis [18]. All patients received pyridoxine 50 mg daily during the whole course antituberculous treatment.

Prophylaxis of deep vein thrombosis with subcutaneous sodium heparin (5000 IU every 12 hours) was given during the hospital admission to all patients. Intravenous dexamethasone was given the first day along with the ATR and was rapidly tapered if a clinical improvement was observed. The typical regimen was dexamethasone 16 mg IV every 6 hours for 3-4 days, dexamethasone 8 mg IV every 8 hours for 3-4 days, and then oral prednisolone 40 mg once daily before hospital discharge. After discharge, prednisolone was reduced 10 mg every two weeks, so corticosteroids were stopped after 8 weeks of hospital discharge. Patients with seizures were treated with a loading oral dose of 800 mg of valproate acid and then 600 mg every 8 hours. If seizures were controlled, a further reduction to 400 mg every 8 hours was performed before hospital discharge. 

### 2.3. Definitions

Designation of patients' community was performed by self-identification of the patients. Scheduled caste community is marginalized in the traditional Hindu caste hierarchy and, therefore, suffers social and economic exclusion and disadvantage. Scheduled tribe community is generally geographically isolated with limited economic and social contact with the rest of the population. Scheduled castes and scheduled tribes are considered socially disadvantaged communities and are supported by positive discrimination schemes operated by the Government of India [19]. Depressed level of consciousness was defined in patients with confusion, disorientation, or coma, but the Glasgow coma scale was not calculated.

### 2.4. Statistical Analysis and Ethics Statement

Statistical analysis was performed using Stata Statistical Software (Stata Corporation Release 11 College Station, TX, USA). The primary endpoint of the study was to evaluate the mortality in patients receiving the standard ATR versus the new ATR, so time-to-event methods were used. Time was measured from ATR initiation to death. Patients who did not die during the study period were censored at their last visit date. Crude and multivariable analyses were performed with Cox regression proportional hazard models. The proportional hazard assumption was assessed performing log-log survival curves based on Schoenfeld residuals [20]. CD4 cell count, sodium, haemoglobin, and albumin were not available in 12, 14, 8, and 10 cases, respectively. To include these patients in the multivariable analysis, missing values were imputed using multiple imputations by chained equation assuming missing at random [21]. In a sensitivity analysis, we also performed a multivariable analysis using only those cases with complete information. The study was approved by the ethical committee of the RDT Institutional Review Board.

## 3. Results

We identified 213 patients with diagnosis of tuberculous meningitis in the VFHCS database. After reviewing all cases, 10 patients were excluded for not meeting the criteria for tuberculous meningitis [15]. Of the 203 patients included in the analysis, 138 received the standard initial ATR and 65 received the new ATR. Baseline characteristics of the patients are presented in Table 1. Median age was 36 years and near one-third were women. One-third of patients belonged to disadvantaged communities, 5% were homeless, and over half were illiterate. 41% had started HIV antiretroviral therapy before the diagnosis of tuberculous meningitis and 20% had a previous episode of tuberculosis. 44% presented to the hospital with depressed consciousness, and the median CD4 lymphocyte count was 107 cells/*μ*L. At presentation, the median sodium, haemoglobin, and albumin were 132 mEq/L, 10.3 g/dL, and 3.4 g/dL, respectively. We did not find statistically significant differences between patients who received the standard ATR and the new ATR (Table 1). Among patients who received the new ATR and did not die during the first admission, the median duration of the new ATR was 7 days (interquartile range (IQR), 6 to 9; range 3 to 24). Weight was measured in 102 patients who received the standard ATR and in 50 patients who received the new ATR, and the median weight was 43 kg (IQR 38–48) and 44 kg (IQR 40–50), respectively. Therefore, in patients who had their weight measured, the median dose of isoniazid and rifampicin was 7 mg/kg (IQR 6.3–7.9) and 10.5 mg/kg (IQR 9.4–11.8), respectively, for the group who received the standard ATR and 13.6 mg/kg (IQR 12–15) and 20.5 mg/kg (IQR 18–22.5), respectively, for the group who received the new ATR. In the group who received the standard ATR, the median followup was 7.5 months (IQR 0.4–23.5) and, during the first 12 months after the initiation of the ATR, 70 (51%) patients died, 5 (4%) were lost to followup, and 63 (46%) patients were alive. The group who received the new ATR had a smaller follow-up period, median 4.2 months (IQR 1.9–9.3), because the treatment was implemented in the hospital more recently. During the study period, 19 (29%) patients died, 43 (66%) attended the hospital within three months before the end of the study period, and 3 (5%) did not attend the hospital within three months before the end of the study period. 

**Figure 1:**
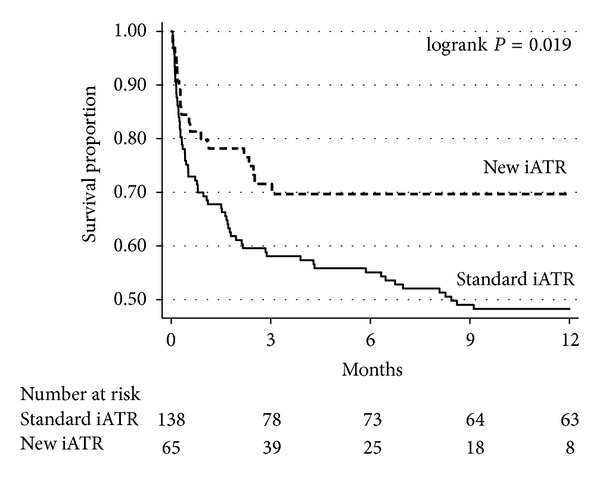
Kaplan-Meier survival estimates by the initial antituberculous regimen of 203 HIV patients with tuberculous meningitis. iATR, initial antituberculous regimen.

Figure 1 presents the Kaplan-Meier survival estimates by initial ATR. The study involved 2070.4 person-months. Patients who received the new ATR had a significant lower mortality than patients who received the standard ATR (*P* value = 0.019). The estimated absolute mortality reduction of the new ATR at 1, 3, 6, and 12 months was 10.5% (95% CI, −2 to 23), 13.5% (95% CI, −0.005 to 27.4), 14.6% (95% CI, 4.6 to 28.8), and 21.5% (95% CI, 7.3 to 35.7), respectively.

**Figure 2:**
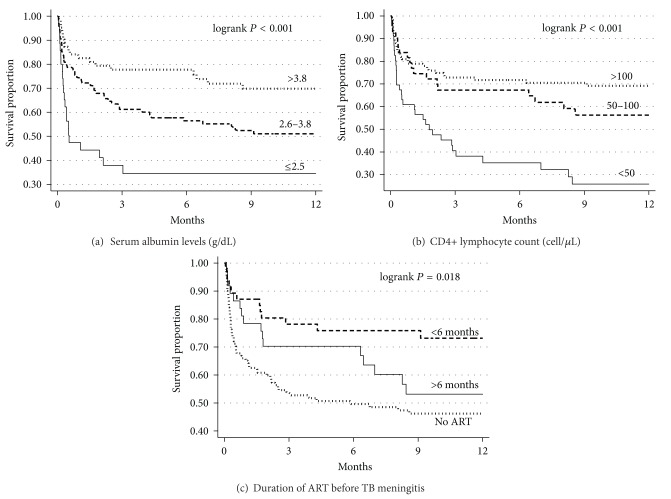
Kaplan-Meier survival estimates by baseline serum albumin levels (a), CD4 lymphocyte count (b), and duration of antiretroviral therapy (ART) of 203 HIV patients with tuberculous meningitis (c). ART, antiretroviral therapy; TB, tuberculous.

Crude and multivariable analyses of factors associated with mortality are presented in Table 2. Compared to the new ATR, the standard ATR had an adjusted hazard risk of mortality of 2.05 (95% CI, 1.21–3.42). In a sensitivity analysis including only those patients who had no missing values, the association between mortality and the standard ATR was also statistically significant. Other factors found to be associated with mortality were not being on antiretroviral therapy versus being on antiretroviral therapy <6 months, having a CD4 lymphocyte count <50 cells/*μ*L and having a serum albumin <3.8 g/dL. Kaplan-Meier survival estimates by serum albumin levels, CD4 lymphocyte count, and duration of antiretroviral therapy before tuberculous meningitis are presented in Figure 2.

## 4. Discussion 

In this study performed in a “real-life” resource-limited setting, the use of an initial ATR with better CSF drug penetration was associated with an estimated absolute mortality reduction at 12 months of 21.5%, and after adjusting by other variables, patients who received the standard ATR had two times higher risk of mortality than patients who received the new ATR. If these findings are confirmed by other studies, the result of this study could change the initial management of tuberculous meningitis in HIV infected patients.

The results of this study also add to the literature by showing new prognostic factors in HIV-related tuberculous meningitis that can be used in resource-limited settings. Patients who were on antiretroviral therapy at the time of tuberculous meningitis had a significant lower mortality than patients who had not started antiretroviral therapy. This may be explained by the fact that patients who were on antiretroviral therapy might have been asymptomatic at the moment of initiating HIV treatment. In these cases, tuberculous meningitis might have been unmasked after the immunological recovery due to antiretroviral therapy [22], suggesting that these patients had a less advanced form of tuberculous meningitis than those who were not on HIV treatment. In accordance with previous studies [23], patients with low CD4 counts were at a higher risk of mortality. But perhaps what is more interesting for resource-limited setting is that having low levels of serum albumin was strongly associated with mortality. Having low serum albumin levels has been associated with a higher risk of death in HIV-related tuberculosis and in patients initiating antiretroviral therapy [24, 25]. The present study shows that serum albumin can be used as a low-cost prognostic factor in HIV-related tuberculous meningitis.

The study has some limitations. Unlike clinical trials, observational studies can be biased due to unknown confounders. This is a retrospective study reflecting the routine clinical practice in a resource limited setting. Although we used the definition of possible tuberculous meningitis as the minimum criteria for patients to be included in the analysis [9, 15], the study was not designed to correctly classify cases as possible, probable, or definite tuberculous meningitis. We did not perform mycobacterial culture and drug sensitivity testing, so we do not know the proportion of patients who had drug resistant tuberculosis in each group. The Glasgow coma scale of patients was not calculated. Thus, we do not know whether patients who received the standard ATR had a more depressed level of consciousness than patients who received the new ATR. However, although not statistically significant, the proportion of patients with depressed consciousness was higher in the new ATR group (50.8% versus 40.6, *P* value = 0.17). The new ATR was well tolerated and we did not observe any severe adverse drug reaction. However, the study was not designed to detect adverse drug reactions, so the safety of the new ATR must be evaluated in further studies. The follow-up period of the group who received the new ATR was smaller than the one of the group who received the standard ATR because the new ATR was implemented more recently in our hospital. However, right censoring was corrected in multivariable analysis using Cox proportional hazard models. In summary, our findings must be interpreted with caution, and new studies, preferably randomized clinical trials, may be needed before adopting this new ATR into the clinical practice. 

## 5. Conclusions 

The results of this study indicate that improving the CSF drug penetration of the initial treatment of tuberculous meningitis can have a beneficial effect on survival in HIV infected patients. If these findings are confirmed by other studies, the result of this study could change the clinical management of tuberculous meningitis in HIV infected patients.

## Figures and Tables

**Table 1:** Baseline characteristics and differences between patients who received the standard antituberculous regimen and those who received a new antituberculous regimen with better cerebrospinal fluid penetration.

	Overall	Standard iATR	New iATR	*P*-value
	*N* = 203	*N* = 138	*N* = 65	
Continuous variables	Median (IQR)	Median (IQR)	Median (IQR)	Wilcoxon rank-sum test

Age (years)	36.1 (30.3−43.4)	36.5 (30.9−45)	36 (30−42)	0.421
CD4 count (cells/*μ*L)	107 (52−173)	108 (51.5−186)	107 (52−163)	0.744
Haemoglobin (g/dL)	10.3 (8.5−12)	10.5 (8.6−12.1)	9.7 (8.1−11.3)	0.182
Albumin (g/dL)	3.4 (2.7−4)	3.5 (2.8−3.9)	3.3 (2.6−4)	0.347
Sodium (mEq/L)	132 (126.8−136)	132.9 (126.9−136.7)	131.6 (126.8−134.9)	0.386

Categorical variables	*N* (%)	*N* (%)	*N* (%)	Chi^2^ test

Gender				0.69
Female	60 (29.56)	42 (30.66)	18 (27.69)	
Male	143 (70.44)	95 (69.34)	47 (72.31)	
Disadvantaged community				0.703
No	140 (68.97)	94 (68.12)	46 (70.77)	
Yes	63 (31.03)	44 (31.88)	19 (29.23)	
Homeless				0.579
No	193 (95.07)	132 (95.65)	61 (93.85)	
Yes	10 (4.93)	6 (4.35)	4 (6.15)	
Illiterate				0.75
No	97 (47.78)	67 (48.55)	30 (46.15)	
Yes	106 (52.22)	71 (51.45)	35 (53.85)	
Depressed consciousness				0.172
No	114 (56.16)	82 (59.42)	32 (49.23)	
Yes	89 (43.84)	56 (40.58)	33 (50.77)	
Previous tuberculosis				0.407
No	163 (80.3)	113 (81.88)	50 (76.92)	
Yes	40 (19.7)	25 (18.12)	15 (23.08)	
Months on ART				0.755
>6 months	37 (18.23)	27 (19.57)	10 (15.38)	
<6 months	47 (22.15)	32 (23.19)	15 (23.08)	
No ART	119 (58.62)	79 (57.25)	40 (61.54)	

ART: antiretroviral therapy; iATR: initial antituberculous regimen; IQR: interquartile range.

**Table 2:** Crude and multivariable Cox regression analyses of factors associated with mortality in HIV patients with tuberculous meningitis.

	Crude analysis		Complete case MA		Multiple imputation MA	
			*N* = 176		*N* = 203	
	HR	95% CI	aHR	95% CI	aHR	95% CI
iATR						
New	1		1		1	
Standard	1.82*	(1.095–3.020)	1.88*	(1.081–3.261)	2.05*	(1.209–3.462)
Age (years)						
<35	1.06	(0.711–1.573)	1.16	(0.695–1.922)	1.09	(0.692–1.729)
>35	1		1		1	
Gender						
Female	1.05	(0.683–1.601)	0.93	(0.538–1.594)	0.85	(0.514–1.405)
Male	1		1		1	
Disadvantaged community						
No	1		1		1	
Yes	1.59*	(1.052–2.399)	1.19	(0.717–1.963)	1.27	(0.811–1.974)
Homeless						
No	1		1		1	
Yes	0.89	(0.361–2.201)	1.05	(0.382–2.874)	1.02	(0.378–2.766)
Illiterate						
No	1		1		1	
Yes	1.31	(0.877–1.954)	1.12	(0.662–1.889)	1.18	(0.736–1.881)
Depressed consciousness						
No	1		1		1	
Yes	1.36	(0.913–2.021)	1.21	(0.751–1.939)	1.30	(0.852–1.987)
Previous tuberculosis						
No	1		1		1	
Yes	1.09	(0.657–1.792)	1.54	(0.795–2.966)	1.50	(0.845–2.678)
Months on ART						
>6 months	0.63	(0.367–1.098)	0.82	(0.403–1.678)	0.76	(0.399–1.439)
<6 months	0.50*	(0.291–0.852)	0.59	(0.310–1.142)	0.56*	(0.311–0.995)
No ART	1		1		1	
CD4 count (cells/*μ*L)						
≤50	3.04*	(1.885–4.901)	1.93*	(1.086–3.427)	1.95*	(1.132–3.372)
51–100	1.34	(0.766–2.346)	1.24	(0.665–2.322)	1.27	(0.707–2.279)
>100	1		1		1	
Sodium (mEq/L)						
≤135	1		1		1	
>135	0.58*	(0.358–0.956)	0.66	(0.379–1.133)	0.70	(0.410–1.181)
Albumin (g/dL)						
≤2.5	3.95*	(2.163–7.231)	3.66*	(1.490–9.008)	3.14*	(1.433–6.898)
2.6–3.8	1.92*	(1.139–3.227)	1.95*	(1.029–3.691)	1.92*	(1.093–3.380)
>3.8	1		1		1	
Haemoglobin (g/dL)						
≤9	1.48	(0.973–2.239)	0.78	(0.421–1.428)	0.76	(0.434–1.330)
>9	1		1		1	

*P value < 0.05; aHR, adjusted hazard ratio; ART: antiretroviral therapy; iATR: initial antituberculous regimen; HR: hazard ratio; MA: multivariable analysis.
